# Management of Macroglossia Secondary to Beckwith-Weidmann Syndrome in a Pediatric Patient: A Case Report

**DOI:** 10.7759/cureus.46579

**Published:** 2023-10-06

**Authors:** Dhruvi Solanki, Punit Fulzele, Nitin Bhola

**Affiliations:** 1 Pediatric Dentistry, Sharad Pawar Dental College and Hospital, Datta Meghe Institute of Higher Education and Research, Wardha, IND; 2 Oral and Maxillofacial Surgery, Sharad Pawar Dental College and Hospital, Datta Meghe Institute of Higher Education and Research, Wardha, IND

**Keywords:** pediatric surgery, tongue abnormalities, electocautery, beckwith-wiedemann syndrome, macroglossia

## Abstract

Beckwith-Wiedemann syndrome (BWS) is a rare genetic disorder, distinguished by the following characteristics: macrosomia, macroglossia, abdominal wall deformities such as omphalocele, visceromegaly, hemihypertrophy and elevated risk of developing tumors such as nephroblastoma or hepatoblastoma.

A 2.5-year-old female patient came to the Department of Pediatric and Preventive Dentistry with a complaint of abnormally large tongue along with difficulty in swallowing and slurred speech. On clinical examination, the built of the patient was greater than normal. Intraoral examination revealed an enlarged tongue that led to the inability to close her mouth. Preliminary tests like blood tests, ECG, etc., were done before proceeding further to correct the enlarged tongue surgically under general anesthesia. The patient was intubated nasally, and a keyhole incision pattern was marked on the dorsum of the tongue at the central part. Reduction glossectomy was performed using electrocautery and the two parts were thereafter sutured with 5-0 vicryl sutures. The patient was kept under observation for one week and then discharged. Satisfactory healing was observed. Early diagnosis, close monitoring by healthcare specialists, and a thorough treatment plan that includes speech therapy, food support, and dental care can help manage the issues associated with BWS macroglossia.

## Introduction

Beckwith-Wiedemann syndrome (BWS) is a rare genetic disorder that influences growth and developmental processes. It derives its name from the pioneering physicians who independently identified this disorder during the 1960s, namely Dr. Bruce Beckwith and Dr. Hans-Rudolf Wiedemann. It is characterized by overgrowth and emerges as a manifestation of human imprinting disorder. This complex condition is intricately linked to genetic and epigenetic alterations in chromosome 11p15, a genetic locus that imparts its effects on fetal and postnatal growth processes. These aberrations may either arise sporadically or passed down through generations. According to estimates, there is one afflicted kid for every 10,340 live births. Notably, a substantial portion of BWS cases, ranging from 13% to 20%, presents with cardiac abnormalities. Moreover, an overwhelming majority, around 90%, of BWS patients exhibit the hallmark of macroglossia [1].

This syndrome manifests with a diverse spectrum of clinical presentations. Notably, individuals afflicted with BWS typically exhibit the following distinctive features: Macrosomia, children with BWS frequently develop excessively before and after delivery, and the babies are born significantly larger. Macroglossia manifests as an enlarged tongue, leading to eating and speaking issues. Moreover, it may be accompanied by abdominal wall deformities such as omphalocele. Some individuals with BWS may present with enlarged internal organs, including the liver or kidneys, a condition regarded as visceromegaly. Hemihypertrophy, wherein ear pits or creases may also be seen in some individuals. Additionally, those affected by BWS face an elevated risk of developing tumors such as nephroblastoma or hepatoblastoma [1,2].

BWS treatment and management generally entail addressing the individual symptoms and complications that may emerge. To provide comprehensive care, a multidisciplinary approach is employed, comprising pediatricians, geneticists, surgeons, and other specialists. Regular tumor growth monitoring is a crucial element. The particular treatment approach differs depending on the individual's requirements and symptoms. Furthermore, genetic counseling holds paramount importance, particularly for families with a history of BWS [3].

## Case presentation

**Figure 1:**
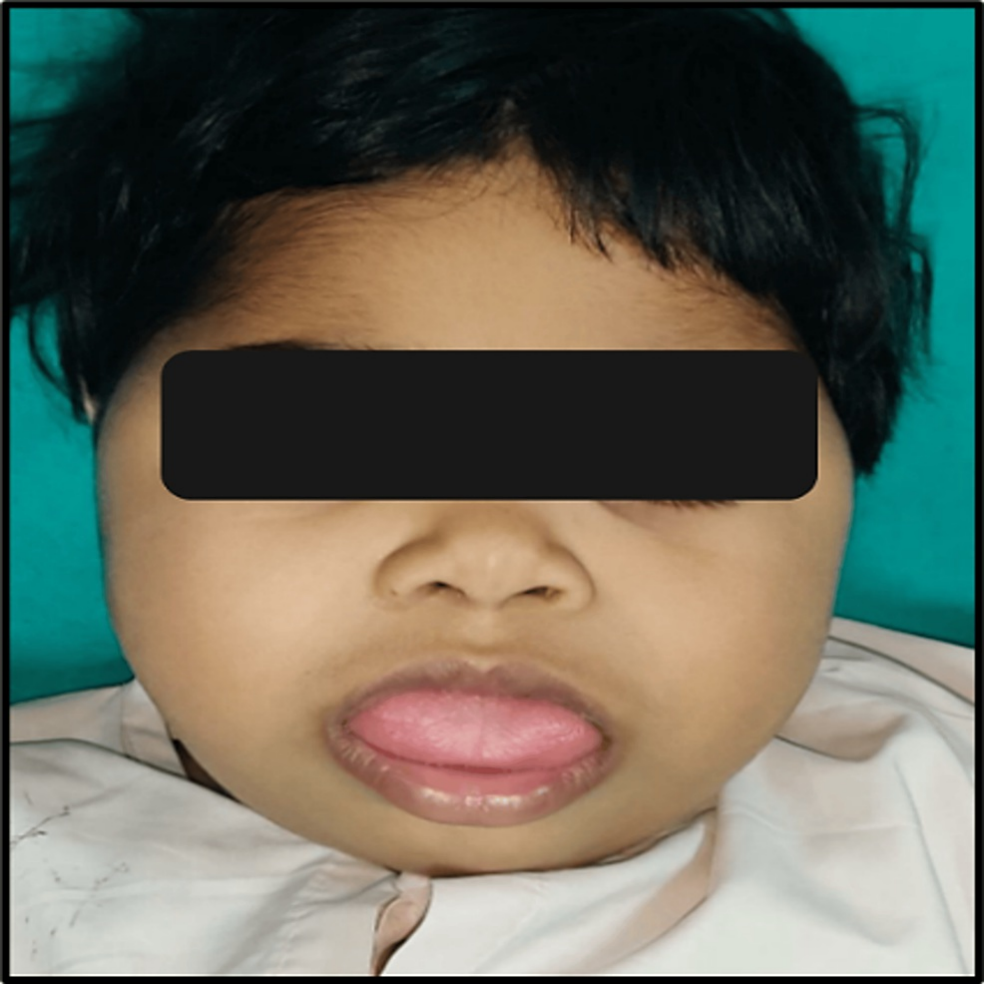
Extraoral image of the patient showing enlarged tongue and open mouth.

A 2.5-year-old female patient of Asian ethnicity, presented to the Department of Pediatric and Preventive Dentistry with a complaint of an abnormally large tongue coupled with challenges in swallowing and impaired speech articulation. A review of the patient's medical history disclosed her as a previously diagnosed case of BWS. Also, the patient had mild cardiomegaly. The patient attained all the developmental milestones as per her age. On clinical assessment, it became evident that the patient's build was larger. Extraorally, no gross facial asymmetry was noted, apart from the lips, which were incompetent with an abnormally large tongue protruding between them (Figure 1).

**Figure 2:**
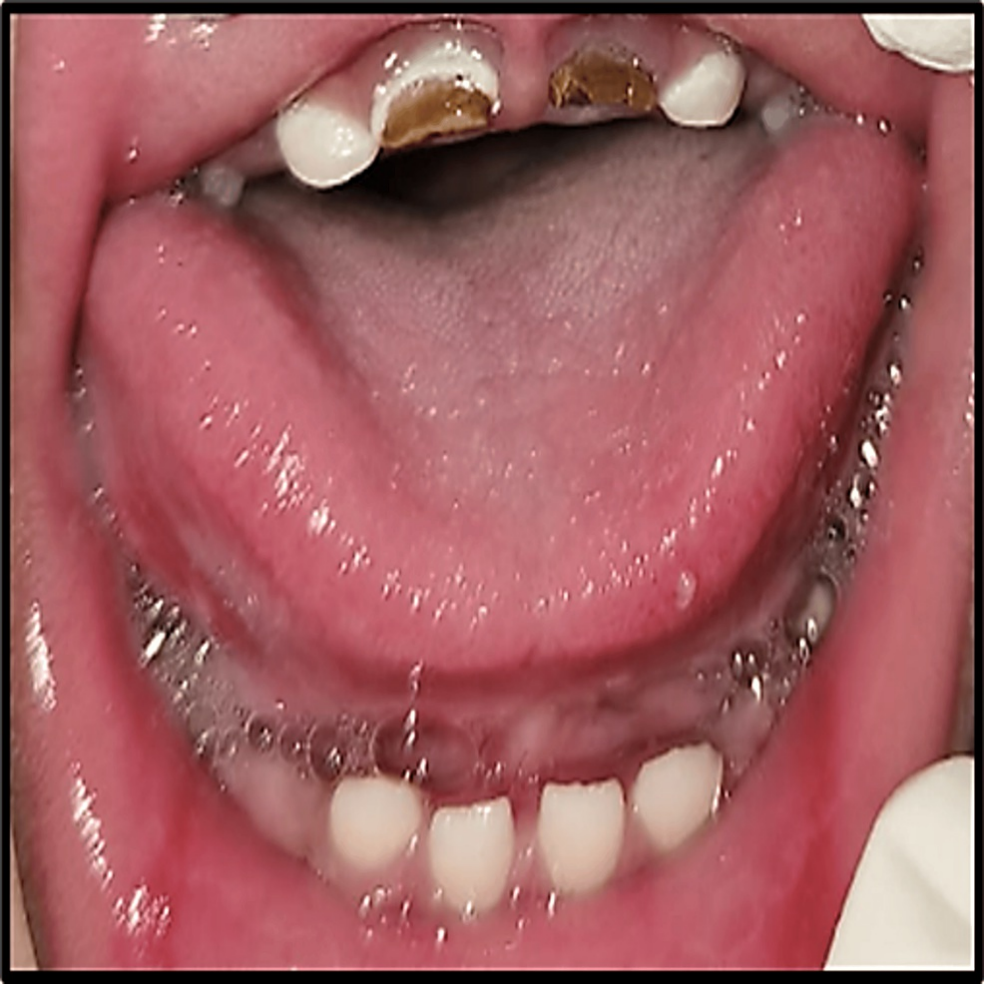
Intraoral examination showing dental status.

This resulted in dribbling of saliva, hindered drinking and swallowing abilities, and also an inability to close her mouth. In both the arches, deciduous incisors, canines, and first molars were present. Maxillary central incisors were grossly carious (Figure 2).

**Figure 3:**
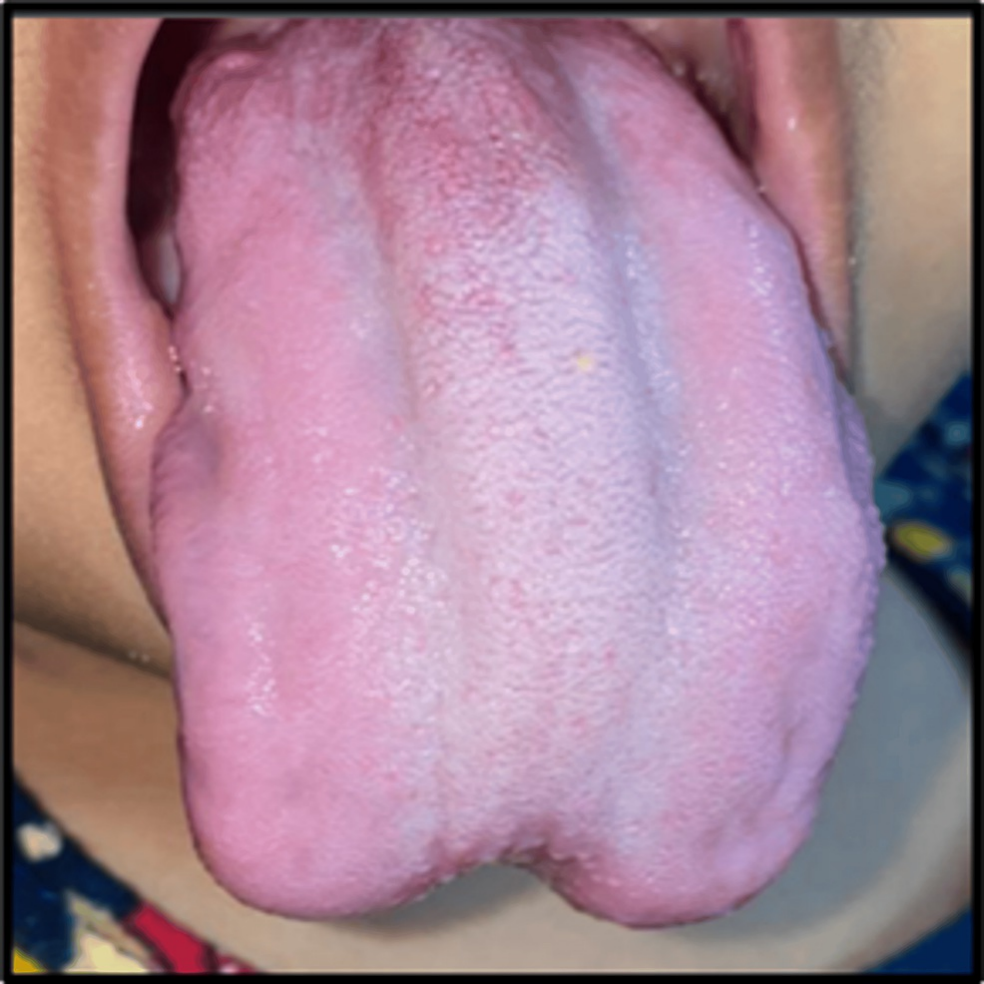
Dorsal aspect of the enlarged tongue

**Figure 4:**
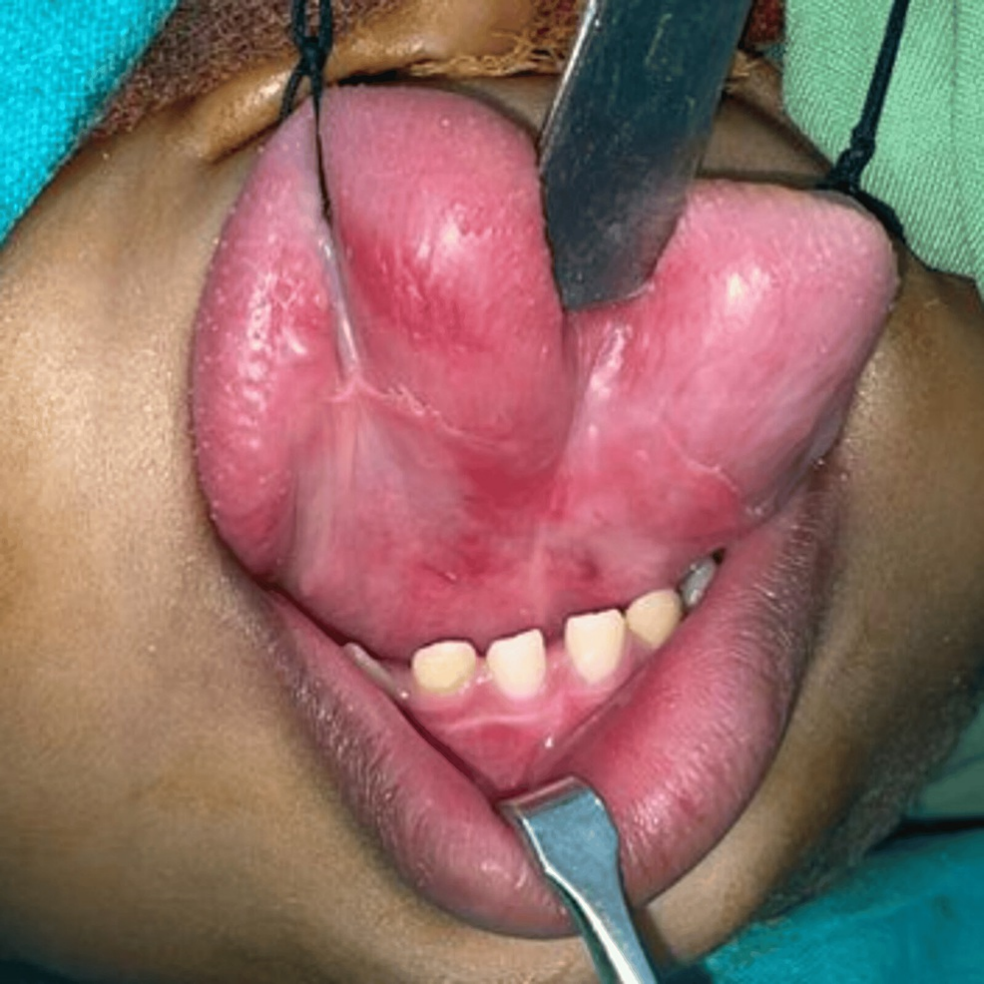
Ventral aspect of the tongue

Similarly, maxillary first molars exhibited dentinal caries. Based on clinical examination, a diagnosis of Macroglossia secondary to BWS and Early Childhood Caries was given (Figures 3, 4). 

**Figure 5:**
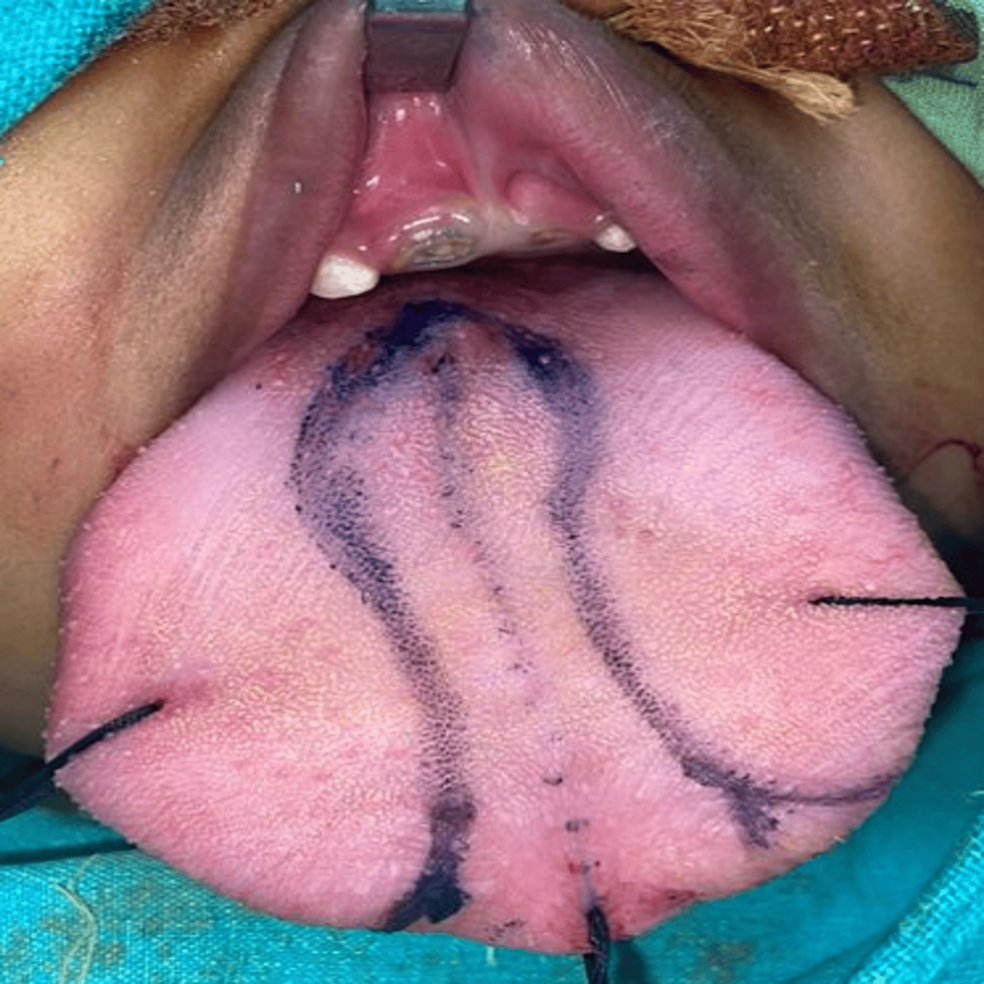
Intra-operative pictures showing keyhole pattern marking on the dorsal aspect of the tongue

Before embarking on surgical intervention to rectify the enlarged tongue, a series of preliminary tests, including blood work and an electrocardiogram, were done to ensure the patient's fitness for the procedure. Informed consent was diligently obtained from the parents before proceeding. The patient was intubated nasally, and a keyhole incision pattern was marked on the dorsum of the tongue at the central part (Figure 5). 

**Figure 6:**
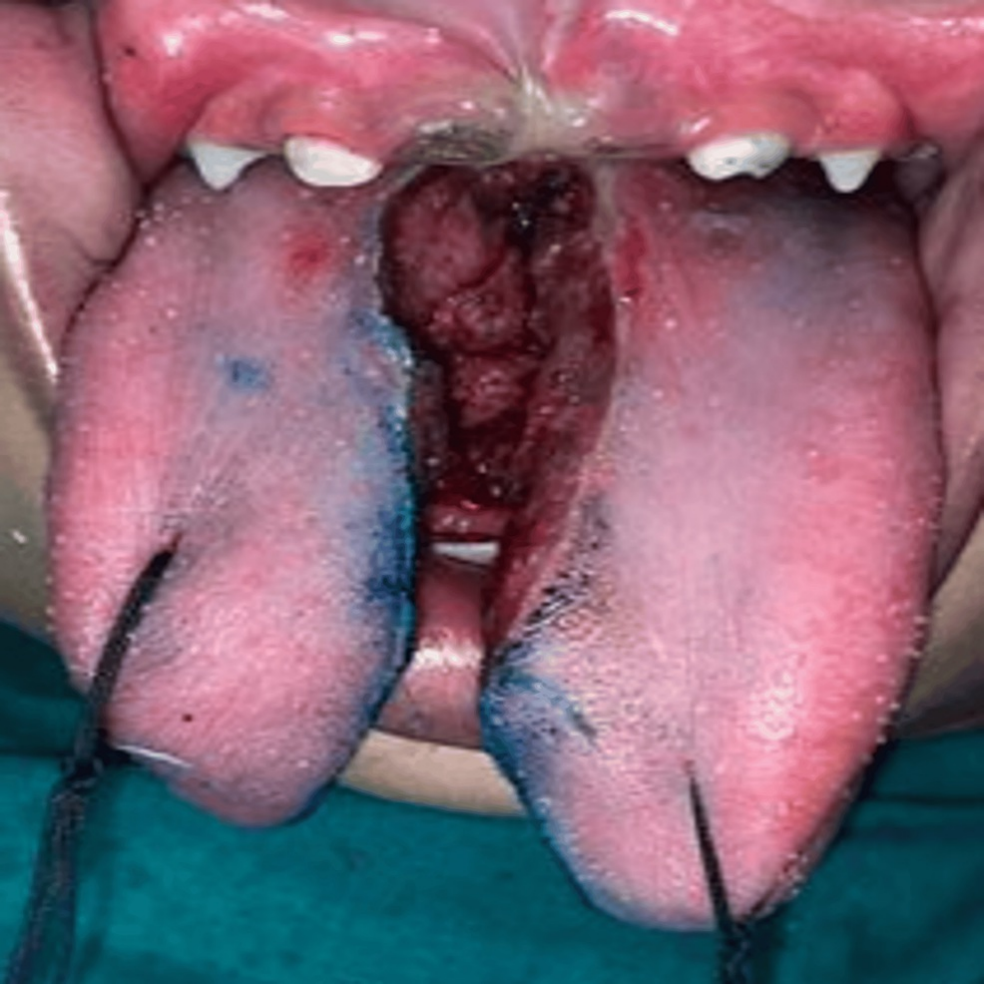
Intra-operative pictures showing subsequent removal of the central marked part of the tongue.

Reduction glossectomy was performed using electrocautery, dividing the tongue into right and left segments (Figure 6). 

**Figure 7:**
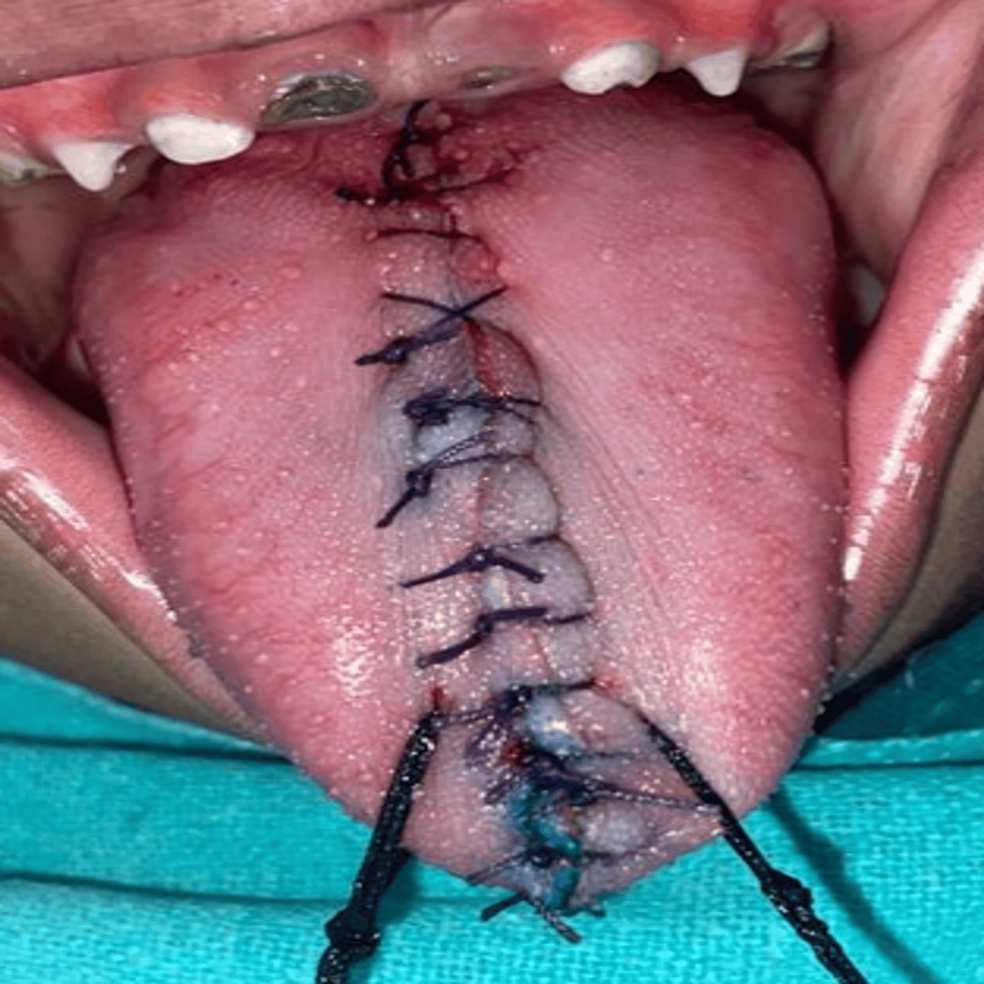
Sutured right and left segments of the tongue: dorsal aspect

**Figure 8:**
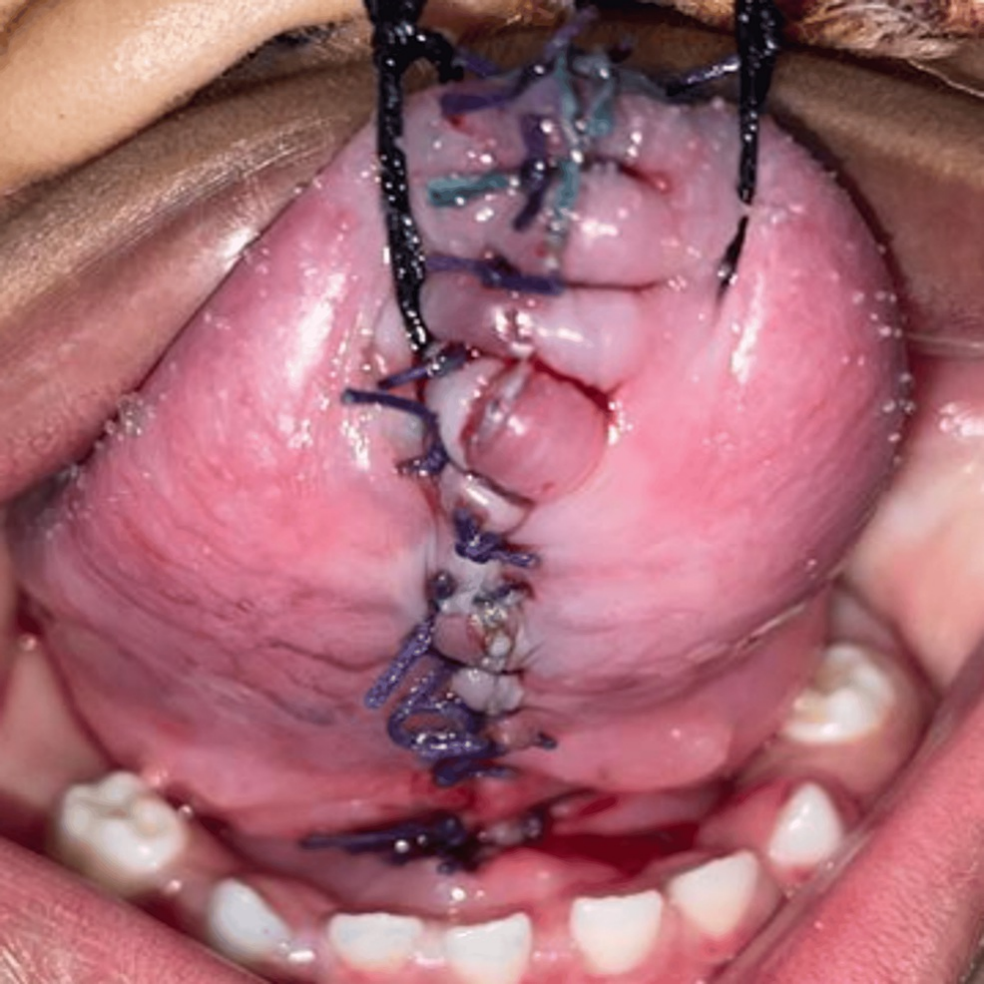
Sutured right and left segments of the tongue: ventral aspect.

Subsequently, the two segments were sutured together using 5-0 vicryl sutures (Figures 7, 8). Healing was uneventful. 

The novelty of this case is that “Macroglossia Secondary to BWS in a Pediatric Patient” is a rare anomaly, and proper clinical diagnosis and treatment is necessary to treat the patient.

## Discussion

Apart from macroglossia and increased body size, another significant facet of BWS encompasses defects in the abdominal wall. These three attributes serve as the primary diagnostic features of BWS. Macroglossia is diagnosed depending on the morphology and tongue protrusion and its impact on various facets like dietary, functional, developmental, and psychological aspects. Recent research has unveiled that macroglossia in BWS histologically displayed actual muscular hyperplasia rather than muscle hypertrophy, which can be owed to an increase in skeletal muscle fiber count without an increase in fiber diameter [2].

The best therapeutic approach for BWS patients with macroglossia is tongue reduction. This intervention serves the critical purpose of mitigating issues such as excessive drooling, difficulties with eating, impaired respiration, and speech and preventing complications related to facial aesthetics and dental occlusion. However, determining the precise timing for surgical intervention remains debatable [3,4]. The timing of the surgical procedure carries significant implications, as both premature and delayed tongue reduction interventions pose potential risks. Early intervention may lead to the regrowth of the tongue or the development of orofacial malformations. Conversely, delaying the procedure may result in occlusion changes and jaw deformities due to the slower pace of tongue development [5]. Due to the slower pace of tongue development, Kopriva and Classen advocated delaying surgery until six months of age [6]. Yamada et al. advised conducting glossectomy in patients up to 1.6 years of age due to occlusion changes and jaw deformity [7]. To have favorable, functional, and aesthetically pleasing results, surgery should be performed before age two as suggested by Wang and Lamfoon [2]. In the case at hand, surgical debulking of the tongue was done at the age of 2.5 years of child to restore the anterior contour. Electrocautery was used to minimize blood loss and maintain a clean field.

Beyond the imperative aspect of enhancing functional capabilities, it is equally essential to consider the aesthetic dimensions, particularly the child's facial appearance, when contemplating the execution of a glossectomy procedure. The goal is to achieve a more balanced and proportionate appearance within the oral cavity and face. During the reduction surgery, hypoglossal arteries must be spared along with the lingual nerve and arteries to retain the taste perception of the tongue. After glossectomy, pediatric patients require specialized postoperative care. This encompasses effective pain management, close vigilant supervision during recovery, and attention to dietary and feeding needs. They may also require speech therapy and rehabilitation interventions to facilitate them to adapt to the changes in tongue functionality and speech patterns. Speech therapists play an instrumental role in assisting children in regaining their ability to speak and swallow effectively [4,7]. In this case, after the surgery, the patient was kept in the PICU where regular monitoring of her vitals was done. Also, the patient was kept on liquid diet for one day followed by soft diet for 15 days. Analgesics and antibiotics were prescribed. The parents were advised to follow meticulous oral hygiene regime for the child and betadine gargles.

## Conclusions

BWS is a rare genetic disorder that can manifest with an array of physical abnormalities and health challenges. Focus must be placed on underlying genetic causes and clinical features of BWS as they are essential for its early diagnosis and effective management. It is crucial to highlight that the severity of macroglossia varies among BWS patients, and not all BWS patients require surgery. Early diagnosis, close monitoring by healthcare specialists, and a thorough treatment plan that includes speech therapy, food support, and dental care can help manage the issues associated with BWS macroglossia.
